# Exploring root canal outcomes and periodontal disease in diabetic patients: A review

**DOI:** 10.6026/9732063002001570

**Published:** 2024-11-30

**Authors:** Abdo Mohammed Mohammed Abdulrazzaq, Ali Mohammed Alyami, Fayez Hamad Al Aqil, Abdullah Mohammed Al-khomsan, Sulaiman Mana Al Hutaylah, Hamad Mohammed Al khamssan, Meshal Hussain Alhammami

**Affiliations:** 1 Department of Preventive Dental Science, Faculty of Dentistry, Najran University, Najran, Saudi Arabia; 2 Dental intern, Faculty of Dentistry, Najran University, Najran, Saudi Arabia

**Keywords:** Root canal treatment, diabetes mellitus, systematic review, apical periodontitis, endodontic-periodontal lesions, Treatment outcomes

## Abstract

This review of 14 studies (2000-2024) highlights a significant link between diabetes and poor root canal outcomes, emphasizing the need for specialized, evidence-based management to improve treatment efficacy in diabetic patients with periodontal diseases.

## Background:

Apical periodontitis (AP), an inflammatory root apex disease, often persists in poorly controlled diabetics due to impaired PMN function and delayed wound healing, leading to more periapical lesions [1]. Poorly controlled diabetes increases susceptibility to oral infections, including apical periodontitis, which correlates with higher endodontic treatment failure rates [2]. Diabetes is a key prognostic factor for root canal outcomes, necessitating better-controlled, long-term clinical trials [3]. Type 2 diabetes is linked to higher apical periodontitis rates and slower healing post-root canal therapy [4]. RCT with apical periodontitis was more prevalent in type 11 diabetics, affecting treatment timing and glycemic control [5]. In diabetics, abnormal glucose levels affect root canal outcomes, leading to more periapical lesions and complications [6]. Therefore, it is of interest to show that exploring root canal outcomes and periodontal disease in diabetic patients underscores the need for tailored treatment strategies, as diabetes may impair healing, increase infection risk and necessitate closer monitoring for complications.

## Materials and Methods:

This review examines the evidence on root canal treatment and periodontal diseases in diabetic patients.

## Articles identification:

A systematic PRISMA-based search in PubMed, ScienceDirect and Cochrane identified relevant studies on diabetes, root canal and periodontal disease.

## Study screening:

The study selection followed PRISMA, with duplicates removed and full-text articles evaluated by two authors for inclusion.

## Inclusion:

The review analysed studies from 2000-2024 on root canal treatment and periodontal diseases in diabetic patients.

## Exclusion criteria:

Database results were screened, duplicates removed with EndNote and titles/abstracts reviewed to include relevant studies only.

## Data extraction process:

Two investigators independently extracted data, resolving conflicts through consensus.

## Quality assurance and bias evaluation:

Two authors assessed bias risk using the Hoy tool, focusing on key factors [7].

## Ethics and dissemination:

Since this is a systematic review of published literature, no ethical approval was required. Findings were published and presented at conferences.

## Results:

**Figure 1:**
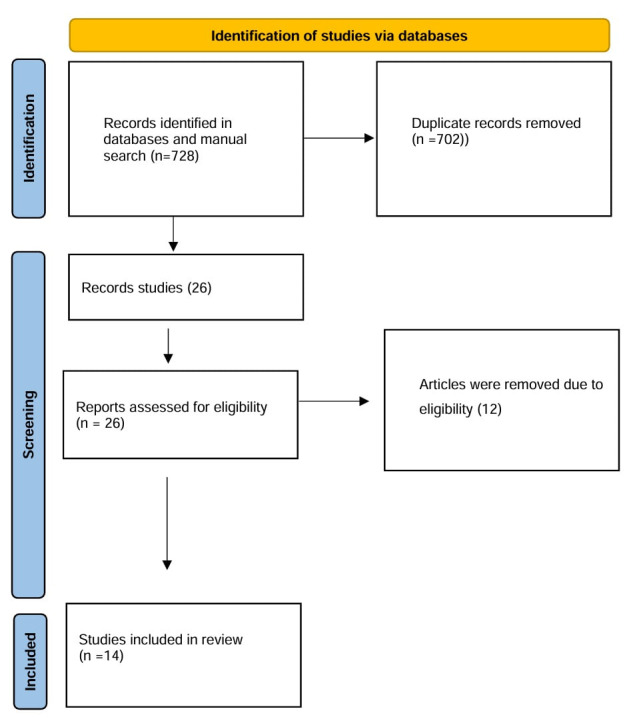
PRISMA flowchart Study selection process for endodontic treatment in diabetics with apical periodontitis.

A search identified 728 studies; 702 were duplicates. After reviewing, 12 studies were excluded and 14 studies met the inclusion criteria for the final analysis. (Figure 1), (Table 1) and (Table 2).

## Explanation:

Smadi (2017) found higher AP prevalence, ET and AP/ET ratio in diabetics (13.5% vs 11.9%, p = 0.001), with poorly controlled DM showing significantly higher AP lesions (p = 0.001). Karolina *et al.* (2022) reported diabetics had higher AP prevalence post-RCT (OR = 1.51, P < .01) and a threefold increased risk of AP (OR = 3.38, P < .01). Juan *et al.* (2016) observed higher RFT prevalence with RPLs in diabetics (OR = 1.42, 95% CI = 1.11-1.80, P = 0.0058). Manuele *et al.* (2014) found no significant differences in success rates (62% test group, 80% control group, p > 0.05). Selen Nihal *et al.* (2019) showed significant differences in AP and cardiovascular disease between DM and control groups (p < 0.05), but no differences within DM subgroups (p > 0.05). José López *et al.* (2011) reported higher AP prevalence (OR = 3.9, P = .002) and more root-filled teeth (OR = 2.3, P = .043) in diabetics. Francisco *et al.* (2020) found T1DM patients had significantly higher RCT (OR = 10.435, P = .000) and AP prevalence (OR = 3.508, P = .011). Patrícia *et al.* (2012) reported higher AP prevalence in untreated diabetic teeth (10%) vs nondiabetic (7%, P = .03).

## Analysis:

Gupta *et al.* (2020) reported higher AP prevalence in diabetics (OR = 1.42) and clinical studies (OR = 6.36). Liu *et al.* (2023) found diabetics had higher AP prevalence post-RCT (OR = 1.51, P < .01), with increased risk at the patient level (OR = 3.38, P < .01) and subgroup analysis showed significance (P < .05). Arya *et al.* (2017) observed reduced periapical scores post-treatment, with diabetics showing less healing (43%) vs. non-diabetics (80%) at 12 months (P < .05). Laukkanen *et al.* (2019) found RCT success rates were lower in DM patients (73.2%) vs. controls (85.6%) (P = 0.043). José *et al.* (2021) reported lower RCT success in diabetics (P < .001). Animal studies by Ashraf *et al.* (2003) showed increased glycemia (P < .001), AP area (P < .05) and lower VEGF (P < .05). Diabetes history was associated with reduced RCT success (P < .01), highlighting the need for tailored endodontic approaches.

## Discussion:

The literature consistently shows a significant association between diabetes mellitus, particularly type 2 diabetes (T2DM) and increased prevalence of apical periodontitis (AP). Diabetic patients, especially those with T2DM, experience higher rates of AP compared to non-diabetics, as evidenced by cross-sectional studies (*e.g.*, Smadi, 2017; Selen Nihal *et al.* 2019) and systematic reviews (Karolina *et al.* 2022; Juan *et al.* 2016). This highlights the need for specialized endodontic care for diabetic patients. Diabetes also affects endodontic treatment outcomes, with studies indicating higher rates of root canal treatment (RCT) failure in diabetics. Research by José *et al.* (2011) and Francisco *et al.* (2020) shows a higher incidence of AP in root-filled teeth among diabetics. Further studies by Gupta *et al.* (2020) and Liu *et al.* (2023) emphasize compromised immune response and chronic inflammation in diabetes, contributing to higher rates of radiolucent periapical lesions. These findings are consistent across diverse regions, including Jordan, Brazil, Lithuania, Portugal and Turkey, reinforcing the robust association between diabetes and AP. Studies using various methodologies converge on similar conclusions regarding the negative impact of diabetes on endodontic outcomes. Diabetes also impairs periapical healing, as demonstrated by Arya *et al.* (2017) and Laukkanen *et al.* (2019), with reduced healing and lower success rates in diabetic patients. Additionally, studies like Ashraf *et al.* (2003) show that diabetic patients with endodontically treated teeth have a higher prevalence of periodontal disease, further complicating treatment outcomes. The clinical implications call for tailored care, including frequent follow-ups, patient education and potentially specialist referrals. However, research limitations, including confounding comorbidities and lack of standardized treatment protocols, suggest the need for more controlled studies and comprehensive patient assessments to improve endodontic care in diabetics.

## Conclusion:

This research clarified the adverse effects of root canal treatment and the increased Incidence of apical periodontitis (AP) in diabetic patients. By integrating and critically assessing existing evidence, the study identified key knowledge gaps and provided valuable insights into the challenges diabetic individuals face during root canal procedures. This synthesis was essential in developing evidence-based guidelines for managing diabetic patients needing root canal treatment. The findings underscore the necessity of customized treatment planning, including risk assessment, tailored treatment plans, comprehensive pre- and post-treatment care, on-going education and further research. Additionally, they emphasize the importance of addressing diabetes-specific complications to enhance clinical outcomes.

## Figures and Tables

**Table 1:** The review included studies comparing the prevalence of Apical Periodontitis (AP) in diabetic and non-diabetic patients undergoing root canal treatment

**Author (Publication year)**	**Location**	**Design**	**Sample size**	**Aim**	**Main findings**
Smadi, 2017 [8].	Jordan	Cross-sectional	291	Compared apical periodontitis incidence in diabetic and non-diabetic endodontic patients	Apical periodontitis was more common in diabetic endodontic patients.
Karolina *et al.* 2022 [4].	Lithuania	Systematic review	15810	Connection between apical periodontitis, root canals, diabetes.	Significant relationship between apical periodontitis, diabetes endodontic patients.
Juan *et al.* 2016 [9].	Brazil	Systematic review and meta-analysis	1593	Reviewed link between diabetes and periapical lesions.	Diabetes linked to increased periapical radiolucency prevalence.
Manuel *et al.* 2014 [10].	Portugal	Retrospective	737	Diabetes impact on periapical tissues, endodontic success.	Increased apical periodontitis prevalence in diabetic patients. with endodontic treatment.
Selen Nihal *et al.* 2019 [11].	Turkey	Cross-Sectional	129	Evaluation apical periodontitis prevalence in diabetic endodontic patients.	Apical periodontitis, bone destruction higher in diabetics.
José López- *et al.* 2011[5].	Spain	Cross-sectional	100	Radiographic evaluation of apical periodontitis in diabetics.	Apical periodontitis was more prevalent in endodontically treated patients with type II diabetes.

**Table 2:** The review included six studies analyzing the negative impact of diabetes mellitus on root canal treatment outcomes.

**Author (Publication year)**	**Location**	**Design**	**Sample size**	**Aim**	**Main findings**
A Gupta *et al.*2020 [2].	Faridabad	Systematic review.	773	Link between DM and periapical lesions prevalence in RCT.	Diabetes significantly associated with periapical radiolucency in root-filled teeth.
Xinyue Liu *et al.*2023 [14].	China	A Meta-Analysis.	1087	Association between DM and AP prevalence post- RCT treatment.	Diabetes may increase apical periodontitis risk in endodontically treated teeth.
Suman Arya *et al.*2017 [15].	India	Prospective.	60	Effect of periapical healing in diabetic patients assessed.	Diabetes mellitus may negatively impact the outcome of endodontic treatment.
Laukkanen *et al.*2019 [16].	Finland	Cross-Sectional	504	Assessed systemic health and tooth factors on root canal outcomes.	Diabetes reduces root canal success, especially with apical periodontitis.
José P *et al.*2021 [17].	Portugal	A retrospective observational study	100	Assessed the association between root canal treatment outcome and diabetes mellitus.	Diabetes is a risk factor affecting the success of root canal treatment.
Ashraf F *et al.*2003 [6].	USA	Univariate and multivariate analyses	824	Assessed periodontal disease impact on endodontic outcomes in diabetics.	Diabetic patients show increased periodontal disease in endodontic-treated teeth.
